# Vinte anos da publicação da regulamentação de preços de medicamentos no Brasil: momento de atualizar?

**DOI:** 10.1590/0102-311XPT029524

**Published:** 2024-09-16

**Authors:** Valdete Aparecida de Melo, Rafael Santos Santana, Dayani Galato

**Affiliations:** 1 Programa de Pós-graduação em Assistência Farmacêutica, Universidade Federal de Santa Catarina, Florianópolis, Brasil.; 2 Universidade de Brasília, Brasília, Brasil.

**Keywords:** Preço de Medicamento, Acesso a Medicamentos Essenciais e Tecnologias em Saúde, Indústria Farmacêutica, Consulta Pública, Legislação Farmacêutica, Drug Price, Access to Essential Medicines and Health Technologies, Drug Industry, Public Consultation, Pharmacy Legislation, Precio de Medicamento, Acceso a Medicamentos Esenciales y Tecnologías Sanitarias, Industria Farmacéutica, Consulta Pública, Legislación Farmacéutica

## Abstract

Os critérios para definir os preços de medicamentos no Brasil estão previstos na *Resolução CMED nº 2/2004* da Câmara de Regulação do Mercado de Medicamentos. Os preços estipulados influenciam o mercado privado e público, o que torna desafiador a revisão de políticas de preços devido a necessidade de harmonizar interesses sociais e econômicos. Uma proposta de revisão dessa Resolução foi disponibilizada por meio da *Consulta Pública SEAE nº 2/2021* da Secretaria de Advocacia da Concorrência e Competitividade/Ministério da Economia, porém, até o momento sem publicação dos resultados consolidados até o momento. Recomendações recentes da Organização Mundial da Saúde em relação à adoção de diferentes limiares para definição de preços de medicamentos são adotadas nessa Resolução, embora essa tenha sido publicada há 20 anos. Com o objetivo de interpretar e descrever o alinhamento e os possíveis avanços e retrocessos nos textos legais relacionados à regulação de preços de medicamentos, foi utilizado o método da pesquisa documental analítica-descritiva, de cunho exploratório. Como resultado, foram mantidas a lista de países referência para conferência de preço internacional e os limiares de referenciamento interno e externo de preços. As omissões normativas da Resolução permanecem na Consulta Pública, como a ausência de critérios para precificar radiofármacos, terapias avançadas e medicamentos sem preço internacional, e sem comparadores no mercado brasileiro para revisar preços e transpor preço provisório para definitivo. Um ponto crítico foi a criação de bônus de 35% acima do preço estipulado para medicamentos que apresentem benefício clínico adicional sem, contudo, definir contornos claros quanto às evidências científicas aceitáveis para a comprovação desse benefício. Em suma, poucos avanços foram percebidos na Consulta Pública.

## Abstract (en)

Criteria for setting medication prices in Brazil are set forth in *CMED Resolution n. 2/2004* of the (Medicines Market Regulation Chamber). The stipulated prices influence the private and public markets, which makes it challenging to review pricing policies due to the need to harmonize social and economic interests. A proposal for reviewing this Resolution was made available through the *SEAE Public Consultation n. 2/2021* of the Competition and Competitiveness Advocacy Secretariat/Brazilian Ministry of Economy; however, so far without publication of the consolidated results. Recent recommendations from the World Health Organization regarding the adoption of different thresholds for setting medication prices are adopted in this Resolution, although it was published 20 years ago. To interpret and describe the alignment, possible advances and setbacks between the legal texts related to medication price regulation, we conducted an analytical-descriptive and exploratory documentary research. As a result, the list of reference countries for international price verification and the thresholds for internal and external price referencing were maintained. The normative omissions of the Resolution remain in the Public Consultation, such as the absence of criteria for pricing radiopharmaceuticals, advanced therapies and medication without international and comparator prices in the Brazilian market, to revise prices and transpose provisional to definitive prices. A critical point was the creation of a 35% bonus above the stipulated price for medication that present additional clinical benefit without, however, defining clear contours as to the acceptable scientific evidence to prove such benefit. In short, few advances were noticed in the Public Consultation.

## Resumen

Los criterios para definir los precios de los medicamentos en Brasil están establecidos en la *Resolución CMED nº 2/2004* de la Cámara de Regulación del Mercado de Medicamentos. Los precios estipulados influyen en el mercado público y privado, lo que dificulta la revisión de las políticas de precios debido a la necesidad de armonizar los intereses sociales y económicos. Una propuesta para revisar esta Resolución se puso a disposición mediante la *Consulta Pública SEAE nº 2/2021* de la Secretaría de Competencia y Promoción de la Competitividad/Ministerio de Economía, sin embargo, hasta el momento no se han publicado los resultados consolidados. En esta Resolución se adoptan recomendaciones recientes de la Organización Mundial de la Salud sobre la adopción de diferentes umbrales para fijar los precios de los medicamentos, aunque fue publicada hace 20 años. Con el objetivo de interpretar y describir el alineamiento, posibles avances y retrocesos, entre los textos legales relacionados con la regulación de precios de medicamentos, se utilizó el método de investigación documental analítica-descriptiva, de carácter exploratorio. Como resultado, se mantuvieron la lista de países de referencia para la verificación de precio internacional y los umbrales para la referenciación interna y externa de precios. Quedan en Consulta Pública las omisiones normativas de la Resolución, como la ausencia de criterios de fijación de precios de radiofármacos, terapias avanzadas y medicamentos sin precio internacional y comparadores en el mercado brasileño, para revisar precios y transponer el precio provisional al definitivo. Un punto crítico fue la creación de una bonificación del 35% sobre el precio estipulado para los medicamentos que presenten un beneficio clínico adicional sin definir, sin embargo, contornos claros sobre las evidencias científicas aceptables para demostrar dicho beneficio. En definitiva, se percibieron pocos avances en la Consulta Pública.

## Introdução

A regulação econômica do mercado de medicamentos permite identificar áreas com necessidades de intervenção. As normas econômicas de regulação para o setor farmacêutico no Brasil estão dispostas na *Lei nº 10.742/2003* ^1^, que também cria a Câmara de Regulação do Mercado de Medicamentos (CMED) e define as suas atribuições, enquanto a *Resolução CMED nº 2/2004* ^2^ (doravante Resolução) estabelece critérios para a definição dos preços de novos produtos e apresentações de medicamentos.

Os preços estabelecidos pela CMED influenciam tanto o mercado privado quanto o público, e segue o modelo de teto de preços (*cap price*) ^3^. Ademais, a estruturação dos preços dos medicamentos ocorre por meio de balizamento entre o referenciamento interno e externo de preços (RIP e REP), conforme as recomendações da Organização Mundial da Saúde (OMS) ^2^ ^,^ ^4^. Outras recomendações desse órgão são a promoção do uso de genéricos e biossimilares e a transparência de preços ^4^.

Embora os medicamentos sejam um dos cuidados cobertos pelo sistema de saúde, os gastos de desembolso direto das famílias com esses itens no período entre 2015 e 2019 girou em torno de 87,7%, e a diferença foi financiada pelo governo ou planos e seguros de saúde. Esse levantamento demonstra que a despesa com medicamentos no Brasil corresponde ao segundo maior peso médio no gasto total com saúde, atrás somente da atenção curativa. Em contrapartida, a cobertura majoritária é realizada pelo governo (56%) em alguns países europeus ^5^. A análise por esse prisma destaca o desafio no campo das políticas de regulação de mercado, tendo em conta a necessidade do medicamento como um bem social e a indústria lucrativa ^6^.

No período de 20 anos de vigência da Resolução, houve mudanças significativas no mercado mundial, a citar as regulamentações sobre patentes, biológicos, terapias avançadas, radiofármacos, além dos altos preços pleiteados por tecnologias com limitadas evidências de eficácia e segurança ^7^ ^,^ ^8^ ^,^ ^9^ ^,^ ^10^.

Com proposta de revisão da Resolução, a *Consulta Pública SEAE nº 2/2021* (doravante Consulta Pública) foi lançada pela Secretaria de Advocacia da Concorrência e Competitividade do Ministério da Economia e esteve aberta para receber contribuições entre julho e setembro de 2021 ^11^. Contudo, até a data de submissão deste artigo, os resultados não estavam disponíveis. Nesse sentido, o objetivo desta publicação foi comparar os textos da Consulta Pública e das regulamentações abarcadas por ela a fim de interpretar e descrever possíveis avanços.

## Método

Esta pesquisa documental analítica-descritiva, de cunho exploratório, decorreu da busca por possíveis inter-relações entre os textos legais consultados ^12^, tendo como pergunta de pesquisa: *As Alterações Propostas pela Consulta Pública SEAE nº 2/2021 Apresentam Avanços Técnico-Normativos em Relação às Atuais Regulamentações de Preço de Medicamentos no Brasil?*

As regulamentações que a Consulta Pública se propõe a revogar são: a Resolução ^2^; o *Comunicado nº 9* ^13^, de agosto de 2016, que trata dos critérios de precificação de biológicos não novos; o *Comunicado nº 10* ^14^, de agosto de 2016, que trata dos prazos de análises de processos; e o *Comunicado nº 4* ^15^, de março de 2017, que trata de transferência de titularidade (Quadro 1). Entre essas regulamentações, somente a Resolução e o *Comunicado nº 9* passaram por alterações técnico-normativas e, por isso, serão detalhados nesta análise.

**Quadro 1:** Descrição das regulamentações de preços de medicamentos no Brasil, incluindo a Consulta Pública.

DOCUMENTOS ANALISADOS	EMENTA	VIGÊNCIA
*Resolução CMED nº 2/2004*	Aprovar os critérios para definição de preços de produtos novos e novas apresentações de que trata o art. 7º da *Lei nº 10.742/2003*	De março de 2004 até os dias atuais
*Comunicado nº 9/2016*	Divulga decisão do CTE sobre os critérios de precificação de medicamentos biológicos não novos	De agosto de 2016 até os dias atuais
*Comunicado nº 10/2016*	Comunica decisão do CTE sobre prazos de análise de recursos ao CTE, reconsiderações, medicamentos liberados e documentos informativos de preço que envolvam casos omissos	De agosto de 2016 até os dias atuais
*Comunicado nº 4/2017*	Divulga entendimentos do CTE referentes à análise de documento informativo de preço de medicamento objeto de transferência de titularidade	De março de 2017 até os dias atuais
*Consulta Pública SEAE nº 2/2021*	Aprova critérios para definição de preços de produtos novos e novas apresentações de medicamentos, de que trata o art. 7º da *Lei nº 10.742/2003*, e dá outras providências	Aberta eletronicamente ao público entre julho e setembro/2021

CMED: Câmara de Regulação do Mercado de Medicamentos; CTE: Comitê Técnico Executivo; SEAE: Secretaria de Advocacia da Concorrência e Competitividade do Ministério da Economia.

Para fins deste artigo, a expressão “regulamentações de preço” se refere ao conjunto de Leis, Resoluções, Comunicados e demais documentos pertinentes publicados pela CMED.

## Resultados

O texto da Consulta Pública manteve inalteradas as orientações mercadológicas para justificar a inclusão dos medicamentos em diferentes categorias de preços, a estruturação de preços por meio do RIP e REP, a lista fixa de países como referência para a busca por preços internacionais e as condições para preço provisório, conforme previsto na Resolução ^2^ ^,^ ^11^.

Ademais, foram sugeridas três novas categorias e os correspondentes critérios para estruturação dos preços: a categoria VII - biológicos não novos ^13^; a categoria VIII - desmembramento da categoria V já existente na Resolução ^2^; e a categoria IX - transferências de titularidade ^15^ (Quadro 2).

**Quadro 2:** Descrição das condições de inclusão de medicamentos nas categorias de preço e dos métodos de estruturação de preços estipulados nas regulamentações vigentes e os sugeridos na Consulta Pública.

REGULAMENTAÇÃO	CATEGORIA DE PREÇO	CONDIÇÕES DE INCLUSÃO	ESTRUTURAÇÃO DE PREÇOS (REGULAMENTAÇÕES VIGENTES)	ESTRUTURAÇÃO DE PREÇOS (*CONSULTA PÚBLICA SEAE Nº 2/2021*)
*Resolução CMED nº 2/2004* (medicamentos sintéticos)	I	Molécula nova no país; patente de molécula; maior eficácia, ou maior segurança, ou redução do custo global de tratamento em relação aos medicamentos existentes no país e que tratam a mesma condição clínica	REP	Condições inalteradas
	II	Molécula nova no país	RIP por custo de tratamento realizado com comparador que atenda a mesma condição clínica; REP como balizador	Condições inalteradas
	III	Nova apresentação; mesma forma farmacêutica; mesma empresa	RIP por média aritmética dos preços das apresentações do mesmo medicamento já comercializado pela empresa; o preço final não poderá ser superior ao preço do medicamento de referência da lista mais atual publicada pela Anvisa	Condições gerais inalteradas. Contudo, houve a inclusão da inaplicabilidade dos genéricos no cálculo do preço
	IV	Nova forma farmacêutica de medicamento com princípio ativo já comercializado pela empresa; nova apresentação na empresa	RIP por preço médio das apresentações dos medicamentos com mesmo princípio ativo, ponderado pelo faturamento de cada apresentação; o preço final não poderá ser superior ao preço do medicamento de referência da lista mais atual publicada pela Anvisa	Condições inalteradas
	V	Nova forma farmacêutica no país; nova associação de princípios ativos já existentes no país; pode apresentar ganhos de eficácia e segurança	Nova forma farmacêutica e nova associação com e sem ganho terapêutico (RIP por custo de tratamento com comparador que atenda a mesma condição clínica, ou RIP por soma das monodrogas e, REP como balizador)	Condições inalteradas para a situação sem ganho terapêutico; a condição de apresentar ganho terapêutico para a nova forma farmacêutica ou para a nova associação foi transferida para a categoria VIII
	VI	Genérico	RIP por deságio de 35% do preço do medicamento referência informado na lista mais atual publicada pela Anvisa	Condições inalteradas
	Caso omisso	Não atende quaisquer condições de inclusão acima descritas	Ausência de critérios definidos	Ausência de critérios definidos
*Comunicado nº 9/2016* (medicamentos biológicos não novos)	Caso omisso	3.1. Medicamentos que comprovem ganho terapêutico	REP	Categoria VII *; RIP por deságio de 20% do preço do medicamento biológico originador; RIP por custo de tratamento com o medicamento biológico originador quando o pleito de preço for para nova apresentação com nova concentração
		3.2. Sem comprovação de ganho terapêutico; novo na lista da empresa	RIP por média do custo de tratamento com medicamento de molécula similar, ponderada pelo faturamento; REP como balizador	
		3.3. Sem comprovação de ganho terapêutico; molécula similar já consta na lista da empresa	RIP por média do custo de tratamento com medicamento de molécula similar da mesma empresa	
		3.4. Novas apresentações; mesma marca comercial já consta na lista da empresa	RIP por média do custo de tratamento com o mesmo medicamento	
N/A	N/A	N/A	N/A	Categoria VIII (nova forma farmacêutica, nova associação ou nova concentração do princípio ativo no país, com benefício clínico adicional): (i) nova associação de princípios ativos no país: RIP por média aritmética dos preços das apresentações dos produtos que compõem a associação, acrescido de bônus de até 35%; (ii) nova forma farmacêutica no país: RIP por custo de tratamento com produtos que o compõem, e REP como balizador, o que for menor, acrescido de bônus de até 35%; (iii) nova concentração de princípio ativo no país: RIP por custo de tratamento com comparador definido, e REP como balizador (o que for menor), acrescido de bônus de até 35%
				Categoria IX **; manutenção do preço da antiga detentora caso a atual empresa não possua medicamento com mesmo princípio ativo; caso a atual detentora tenha medicamentos com mesmo princípio ativo da transferência de titularidade em seu portfólio, o preço será calculado pela média aritmética dos preços destas apresentações

Anvisa: Agência Nacional de Vigilância Sanitária; N/A: não se aplica; REP: referenciamento externo de preços; RIP: referenciamento interno de preços.

Nota: pela *Resolução CMED nº 2/2004*, o preço internacional do medicamento corresponde ao REP, tendo como base a lista fixa dos países: Austrália, Canadá, Espanha, Estados Unidos, França, Grécia, Itália, Nova Zelândia, Portugal e país de origem do medicamento.

* Biológico não novo está previsto no *Comunicado nº 9/2016*;

** Transferência de titularidade está prevista no *Comunicado nº 10/2016*.

Em relação ao *Comunicado nº 9* ^13^, a proposta da Consulta Pública para a estruturação de preço dos biológicos não novos é excluir o REP e eliminar os cálculos por média de custo de tratamento com todos os medicamentos com molécula similar na situação de RIP. A sugestão é precificar somente por deságio de 20% em relação ao preço do biológico originador ou, na situação do pleito de preço ser para uma nova apresentação com nova concentração, aplicar o custo de tratamento com o originador ^11^.

Entretanto, há ausência de definição de critérios para precificar os medicamentos originadores, assim como de critérios para precificar o biológico não novo quando o originador não está presente no Brasil ^11^.

Os casos omissos previstos na Resolução são compreendidos como situações em que as características mercadológicas do medicamento não se enquadram em qualquer dos critérios estabelecidos para categorizá-lo e precificá-lo. Os Comunicados da CMED são considerados atos normativos que tentam complementar ausências na Resolução ao longo dos 20 anos. Contudo, o número de casos omissos ainda deve permanecer alto devido à ausência de inclusão de critérios para precificar os medicamentos sintéticos e biológicos que não possuem preço internacional e nem comparadores no Brasil, os radiofármacos, as terapias avançadas e gênicas, as novas indicações e as “desassociações” (como situação inversa à categoria V), o número de casos omissos ainda deve permanecer alto.

Como falha normativa não diretamente relacionada com a Resolução, foi observado que a lista de medicamentos de referência, que é atualizada e publicada mensalmente pela Agência Nacional de Vigilância Sanitária (Anvisa), possui genéricos e similares. Devido os genéricos entrarem no mercado com um deságio de 35%, todas as categorias de preços que se pautam nos medicamentos de referência como um dos estruturadores de preços podem estar gerando distorções no mercado.

## Discussão

A precificação de “inovações” ^11^ é tema de discussão e publicações que confirmam a pressão por inovação como uma das estratégias do setor produtivo na tentativa de alcançar melhores preços ^16^ ^,^ ^17^. Um estudo realizado no Brasil considerou medicamentos registrados entre 2006-2015 e se amparou nos critérios de inovação recomendados pela Prescrire-Lexchin. Como resultado, foi verificado que apenas 8,2% dos novos medicamentos apresentaram inovação terapêutica que atendem às necessidades locais de saúde. Nesse sentido, observou-se que o setor produtivo reivindica a discussão sobre o preço com o órgão regulador, contudo sem apresentar um plano estratégico de inovação ou que privilegie o desenvolvimento de produtos para necessidades epidemiológicas ainda não supridas da população brasileira ^16^.

Ainda que não haja qualquer inter-relação textual na Consulta Pública entre “inovação” e “benefício clínico adicional”, esses dois temas são debatidos na literatura e envolvem múltiplas perspectivas ^18^. Contudo, quando se trata da oferta do medicamento, da competitividade do setor e da regulação de preços no sentido de tornar a assistência farmacêutica mais efetiva no Brasil conforme recomendam as políticas ^19^, fica evidente o desequilíbrio entre as perspectivas econômica *versus* social na Consulta Pública ao se aplicar 35% de bônus em relação ao REP e/ou RIP. Ainda, é consenso entre pesquisadores que tanto a inovação disruptiva (ou revolucionária) quanto a incremental (ou evolucionária) devem estar pautadas no balanço risco-benefício ^18^, o que é contrariado por meio da proposta de se considerar como benefício clínico “*comodidade posológica, aumento na adesão ao tratamento, redução de resistência antimicrobiana, efeito aditivo ou sinérgico de associações, formulação para populações específicas*” ^11^. Além disso, há uma ausência de contornos claros quanto às evidências científicas para a comprovação do benefício clínico adicional, tendo em conta a graduação da qualidade da evidência.

Sobre a seleção dos países de referência para pautar a busca por preços internacionais, o *Projeto de Lei nº 5.591/2020* ^20^ no Senado Federal sugere alterações para os textos da *Lei nº 10.742/2003* no que se refere à seleção de países. As recomendações são incluir países com compatibilidade socioeconômica e vedar a utilização de preços de medicamentos daqueles que não possuam uma política de regulação de preços e não disponham de sistema público de saúde de acesso universal ^20^.

Os avanços normativos observados em outros países podem ser adotados, como a revisão anual da lista de países referência - em Portugal ^21^, ou a adoção do preço médio entre países referência - na Áustria ^22^. No Canadá, a regulamentação ampliou de sete para 11 países, com exclusão dos Estados Unidos e Suíça e inclusão de seis novos países que têm menores preços ^23^. Na Colômbia, o REP é pautado em 17 países ^24^ e na Itália, entre 24 países europeus ^25^.

Algumas ausências normativas observadas na Resolução que foram mantidas na Consulta Pública são: critérios para determinação de preço de medicamentos que não possuem preços internacionais e nem comparadores no mercado nacional; estabelecimento de condições para revisão periódica de preços considerando a tendência mundial de deságios em determinadas condições, como perda de patente e ciclo de vida ^3^ ^,^ ^10^; e a condução de reanálise de evidências científicas pela ampliação de linha ou de indicações aprovadas. Além disso, tem-se a reestruturação do pensar sobre patentes e a cesta de países, visto que a patente é objeto regional ^18^ e a busca de preços permanece inflexível à limitados locais, sem considerar uma possibilidade de ter em conta os preços praticados em países onde o medicamento não tenha sido patenteado.

Como limitação desta pesquisa, cita-se a ausência de análise da *Lei nº 10.742/2003* e da *Resolução CMED nº 4/2006*, que estipulam, respectivamente, normas para reajuste anual de preços ^1^ e desconto sobre o Preço Fábrica (PF) dos produtos adquiridos pelo governo ^26^. Alguns autores consideram que esses dois temas não estão diretamente relacionados à Resolução, mas influenciam sobremaneira os preços dos medicamentos ^9^ ^,^ ^27^.
